# Editorial: Interplay Between Nutrition, the Intestinal Microbiota and the Immune System

**DOI:** 10.3389/fimmu.2020.01758

**Published:** 2020-08-06

**Authors:** Reinaldo B. Oriá, Nuno Empadinhas, João O. Malva

**Affiliations:** ^1^Laboratory of Tissue Healing, Ontogeny and Nutrition, Department of Morphology, School of Medicine, Institute of Biomedicine, Federal University of Ceara, Fortaleza, Brazil; ^2^CNC-Center for Neuroscience and Cell Biology and CIBB-Center for Innovative Biomedicine and Biotechnology, University of Coimbra, Coimbra, Portugal; ^3^Institute for Clinical and Biomedical Research (iCBR), Center for Innovative Biomedicine and Biotechnology (CIBB) and Institute of Pharmacology and Experimental Therapeutics, Faculty of Medicine, University of Coimbra, Coimbra, Portugal

**Keywords:** gut microbiota, nutrients, inflammation, immune system, intestinal barrier function, autoimmunity

In the last years, evidence is accumulating that the crosstalk between the intestinal microbiota and the immune system is modulated by nutrition and that this modulation can have significant impact on intestinal homeostasis and disease. The interplay between nutrients, gut microbiome, and associated immunomodulation involve a complex network of transcriptional, genetic, and epigenetic programs, much of it remains elusive. Increasing our understanding of how these programs are regulated under different dietary conditions and nutritional states will aid in pinpointing how these intricate factors contribute to the pathogenesis and progression of chronic diseases.

This Research Topic, designed to compile outstanding papers addressing the interplay between nutrients, intestinal microbiota, and the immune system in homeostatic and disease states, could gather original and review articles featuring integrative data on dietary and genetic factors involved in immunoinflammatory conditions such as autoimmunity, obesity, and type1 diabetes.

Yamamoto and Jørgensen's elegant review paper brought new insights on vitamin D deficiency and replenishment effects on systemic autoimmunity with participation of the intestinal microbiota. Vitamin D, as pointed out by the authors, has key actions in the immune system, fostering anti-inflammatory responses, and affecting T cell populations, skewing Th17 to Treg and Breg cells, hence favoring regulatory adaptive immune responses. The authors also concisely reviewed the intestinal dysbiosis effects in autoimmune diseases such as multiple sclerosis, inflammatory bowel disease, rheumatoid arthritis, and systemic lupus erythematosus. The importance of vitamin D for intestinal barrier function and to a healthy gut immune homeostasis is also discussed. Another interesting review from Leocádio et al. addresses the contribution of intestinal dysbiosis to obesity. The authors raised the question whether obesity would be more an infectious disease condition resulting from disrupted intestinal microbiota. This opinion article shed light on how gut microbiota affects body weight and how dietary factors may fuel obesogenic microbiota. Although accumulating evidence from pre-clinical studies points to a key role of the intestinal microbiota in obesity, efficacious microbiota-based therapy against obesity is still missing in clinical settings.

de Sant'Ana et al. studied the influence of inflammasome in high-fat diet (HFD) induced-obesity in caspase 1/11 and Nfrp3 knockout mice. Caspases 1/11^−−/−−^ mice showed increased liver weight and liver steatosis, and changed liver global lipid composition compared to Nfrp3^−/−^ and wild-type mice. Caspase 1/11^−/−^ mice fed a HFD showed increased levels of *Firmicutes, Proteobacteria, Verrucomicrobia*, and lower levels of *Bacteroidetes*, hence a higher Firmicutes/Bacteroidetes ratio than wild-type mice. Altogether these findings support a role for caspases 1/11 in modulating gut microbiota, counteracting obesity.

In another interesting article, Parikh et al. conduct a metagenomic study in 4 and 6-month aged EFAD mice generated from APOE-targeted replacement (either homozygous for human APOE2, APOE3, or APOE4 genes) and 5xFAD crossings. Apolipoprotein E (APOE = gene) is key for cholesterol metabolism and the APOE4 gene is considered a risk factor for Alzheimer's disease. They showed that APOE had even a larger impact than sex or 5xFAD on the intestinal microbiota beta-diversity. APOE4 EFAD mice showed increased intestinal lactobacilli, which may improve intestinal barrier function. In contrast to APOE3 and APOE4, aged APOE2 EFAD mice showed relative abundance of *Ruminococcaceae*, a family of bacteria important for starch digestion in the large intestine and for production of short chain fatty acids (SCFAs) that can promote microglia maturation and function, consequently reducing the risk of AD.

In an elegant paper published in our series, Wu et al. showed that supplementation with a novel-specific low-methoxyl pectin (LMP) attenuated type1 diabetes in non-obese diabetic (NOD) 40-week-old female mice with enriched SCFA-producing bacteria. In addition, LMP supplementation ameliorated intestinal tight junction proteins expression and increased Foxp3^+^ Treg populations and reduced NLRP3 inflammasome in the pancreas and in pancreatic and mesenteric lymph nodes. They performed microbiota transfer after antibiotic-knockdown of the native microbiota that could reverse the previous effects seen with worsened type1 diabetes outcomes. Furthermore, Ishisono et al. published an interesting study featuring the role of dietary pectin in improving TNBS or DSS-induced colitis. Mice were conditioned with the diets with either orange or citrus pectin prior to colitis induction. Orange pectin-fed mice had improved body weight and food intake and better histology with reduced inflammation and decreased frequency of colonic lamina propria CD4RORγt^+^ Th17 cells compared to controls. Pre-treatment of orange pectin also suppressed IL-6 production in macrophages induced by TLR1/2 and TLR4. Altogether, these findings support the benefit of orange pectin supplementation in experimental colitis.

Two enlightening reviews discuss how certain microbial components modulate the host's immune system at the intestinal interface. Russo et al. discuss the immunomodulatory effects of SCFAs and of microbial tryptophan catabolites at the intestinal barrier and address their targets, cell signaling cascades and epigenetic effects. These metabolites are presented as “post-biotics” whose therapeutic potential is discussed in the context of inflammatory bowel diseases (IBD) namely ulcerative colitis (UC) and Crohn's disease (CD). By highlighting the dysbiotic intestinal microbiota as source of post-biotics' imbalance the authors propose nutritional intervention to favor microbiological homeostasis and enrichment of gut post-biotics' and the concept of “immunonutrition” as promising alternative to current approaches to IBD treatment. Finally, Alessandri et al. review the extraordinary interaction between *bifidobacteria* and humans and how evolution has shaped their crosstalk to modulate the immune system since birth or perhaps even earlier. The authors elaborate on how different bifidobacterial-host interactions determine maturation and beneficial modulation of innate and adaptive immunity and on how fermentation of dietary fibers benefits the host immune system and stimulates growth of other SCFAs-producing bacteria. These commensal and symbiotic properties make *bifidobacteria* probiotics of excellence and place them in a gallery of true sentinels of the health of the intestinal ecosystem and host immunity.

In sum, this compilation of papers provides a fine contribution to a better understanding of the interactions of the intestinal microbiota, immune system, and their modulation by nutrients in chronic diseases, which collectively may shed new light to identify novel therapeutic targets for harshly treatable diseases. Intestinal microbiota-based therapy hold promise and we may be looking at next-generation tools to ameliorate or even cure a large spectrum of diseases.

## Author Contributions

All authors have read, approved, and contributed equally for this manuscript.

## Conflict of Interest

The authors declare that the research was conducted in the absence of any commercial or financial relationships that could be construed as a potential conflict of interest.

